# Prediction of kinase inhibitor response using activity profiling, *in vitro* screening, and elastic net regression

**DOI:** 10.1186/1752-0509-8-74

**Published:** 2014-06-25

**Authors:** Trish P Tran, Edison Ong, Andrew P Hodges, Giovanni Paternostro, Carlo Piermarocchi

**Affiliations:** 1 Sanford-Burnham Medical Research Institute, La Jolla, CA 92037, USA; 2 Salgomed Inc., Del Mar, CA 92014, USA; 3 Department of Physics and Astronomy, Michigan State University, East Lansing, MI 48824, USA

**Keywords:** Drug response predictions, Kinase inhibitors, Elastic net regression, High throughput screening, Drug combination therapies

## Abstract

**Background:**

Many kinase inhibitors have been approved as cancer therapies. Recently, libraries of kinase inhibitors have been extensively profiled, thus providing a map of the strength of action of each compound on a large number of its targets. These profiled libraries define drug-kinase networks that can predict the effectiveness of untested drugs and elucidate the roles of specific kinases in different cellular systems. Predictions of drug effectiveness based on a comprehensive network model of cellular signalling are difficult, due to our partial knowledge of the complex biological processes downstream of the targeted kinases.

**Results:**

We have developed the Kinase Inhibitors Elastic Net (KIEN) method, which integrates information contained in drug-kinase networks with *in vitro* screening. The method uses the *in vitro* cell response of single drugs and drug pair combinations as a training set to build linear and nonlinear regression models. Besides predicting the effectiveness of untested drugs, the KIEN method identifies sets of kinases that are statistically associated to drug sensitivity in a given cell line. We compared different versions of the method, which is based on a regression technique known as *elastic net*. Data from two-drug combinations led to predictive models, and we found that predictivity can be improved by applying logarithmic transformation to the data. The method was applied to the A549 lung cancer cell line, and we identified specific kinases known to have an important role in this type of cancer (TGFBR2, EGFR, PHKG1 and CDK4). A pathway enrichment analysis of the set of kinases identified by the method showed that axon guidance, activation of Rac, and semaphorin interactions pathways are associated to a selective response to therapeutic intervention in this cell line.

**Conclusions:**

We have proposed an integrated experimental and computational methodology, called KIEN, that identifies the role of specific kinases in the drug response of a given cell line. The method will facilitate the design of new kinase inhibitors and the development of therapeutic interventions with combinations of many inhibitors.

## Background

The important role of kinases in cancer biology [1] has spurred a considerable effort towards the synthesis of libraries of fully profiled kinase inhibitors, providing a map of the strength of each compound on a large number of its potential targets [2-4]. In particular, a recently published dataset has profiled several hundred kinase inhibitors using a panel of more than 300 kinases [4]. These profiled libraries define a network of interactions between drugs and their kinase targets [5], and represent a valuable resource for the development of new therapies. In this paper, we introduce a novel computational method that incorporates profiled libraries and *in vitro* measurements to predict the response of cells to previously untested drugs. Besides making prediction about the cellular response to drugs, the method identifies critical kinase targets and pathways that are statistically associated to drug sensitivity in a given cell line.

Statistical inference and regression methods in conjunction with gene expression or mutations have been used to identify specific biomarkers associated with an increased sensitivity/resistance to drugs. For instance, the sensitivity to PARP inhibitors of Ewing’s sarcoma cells with mutations in the EWS gene and to MEK inhibitors in NRAS-mutant cell lines with AHR expression have been predicted using analysis of variance and the elastic net method [6] and then experimentally validated [7,8]. In these analyses, the statistical variable associated to drugs was represented by the half maximal inhibitory concentration (IC_50_) in different cell lines. However, besides the IC_50_, there are many other types of information that characterize chemical compounds. These types of information can enhance the statistical analyses and improve the accuracy of predictions. For instance, a method to predict drugs sensitivity in cell lines based on the integration of genomic data with molecular physico-chemical descriptors of the drugs has been recently proposed [9]. Another useful type of information is the residual activity of kinases after interacting with a compound. Kinase profiling, patient genetic profiles, and sensitivity of primary leukemia patient samples to kinase inhibitors were recently used by Tyner *et al.*[10] to identify functionally important kinase targets and clarify kinase pathway dependence in cancer.

In this paper, the residual activity of kinases upon drug interaction is used to make predictions of the cellular response for *in vitro* experiments using an elastic net [6] regression approach. This regression method reduces the number of predictors to a minimum set, providing a clear picture of the kinases involved in the response of cell lines. A primary screen (single drug) and a secondary screen (two-drug combinations) are used as the training set for the regression. The two-drug screening exhibits a broader distribution in the response and provides a good level of predictability. In fact, the model based only on single drug response did not pass the statistical cross-validation test.

We are applying this Kinase Inhibitor Elastic Net (KIEN) method to predict cell viability of a lung cancer cell line (A549) and a normal fibroblast cell line (IMR-90) after drug treatment. We found that the regression can be improved through a logarithmic transformation on the data. Using the results of the regression, we identified a set of kinases that are strongly associated to a selective response of A549 and not IMR-90. Then, a pathway-based enrichment using Reactome [11] revealed ten significant pathways using this set of kinases, including axonal guidance and related semaphorin interactions as top hits.

This paper is organized as follows: Section In vitro screen of the kinase inhibitor library contains the experimental results of the primary and secondary *in vitro* screening corresponding to single drugs and two-drug combinations. These experimental results and residual kinase activity are analyzed with Pearson’s correlation in Section Analysis of correlations. This simple correlation analysis gives a first glance of the kinases that are statistically associated to a significant change in the viability of cancer and normal cell lines. In Section Elastic net regression, we introduce the elastic net approach and we present the results of a leave-one-out cross validation for predictions on single and pairs of drugs. We also present in this section the results obtained using the logarithmic transformation on the variables and a pathway enrichment analysis using Reactome [11]. The Discussion of the results is in Section Discussion, conclusions in Section Conclusions, and Materials and Methods in Section Materials and methods.

## Results

### In vitro screen of the kinase inhibitor library

**Figure 1:**
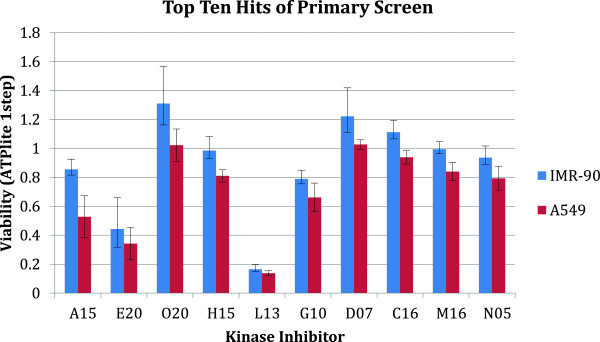
**Primary screen results of the top ten most selective kinase inhibitors.** Drugs are ranked based on the IMR-90 to A549 viability ratio. The 3 digit codes identify the compounds: A15: PDK1/Akt1/Flt3 Dual Pathway Inhibitor (CAS 331253-86-2); E20: Cdk/Crk Inhibitor (CAS 784211-09-2); O20: SU9516 (CAS 666837-93-0); H15: MEK1/2 Inhibitor II (CAS 212631-61-3); L13: PI 3-Kα Inhibitor VIII (CAS 372196-77-5); G10: Fascaplysin, Synthetic (CAS 114719-57-2); D07: Cdk2 Inhibitor II (CAS 222035-13-4); C16: Cdk1/2 Inhibitor III (CAS 443798-55-8); M16: GSK3b Inhibitor XII, TWS119 (CAS 601514-19-6); N05: Reversine (CAS 656820-32-5). The chemical structure of these compounds is given in a Additional file 2.

**Figure 2:**
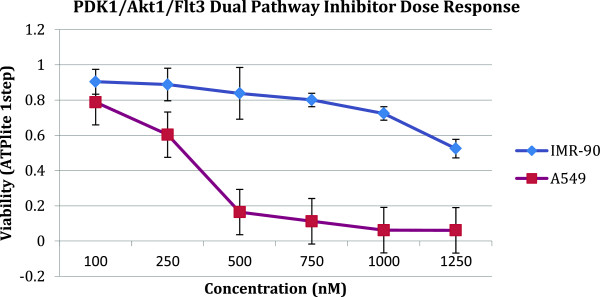
**Dose response curve of PDK1/Akt1/Flt3 dual pathway inhibitor.** Different doses of PDK1/Akt1/Flt3 Dual Pathway Inhibitor were tested to measure the response of A549 to the drug. For the secondary screen we selected 125nM to ensure low toxicity on the normal cell line.

**Figure 3:**
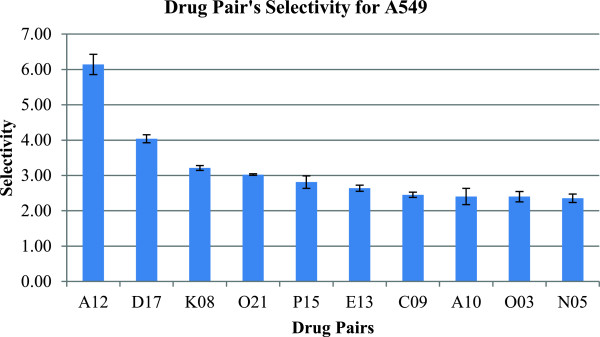
**Secondary screen results of the top ten most selective drugs (1000 nM) when paired with PDK1/Akt1/Flt3 dual pathway inhibitor at 125 nM.** Selectivity is the IMR-90 to A549 viability ratio, as defined in Section 2.1. The 3 digit codes identify the compounds: A12: Alsterpaullone, 2-Cyanoethyl (CAS 852529-97-0); D17: Cdk2/9 Inhibitor (CAS 507487-89-0); K08: K-252a, Nocardiopsis sp. (CAS 97161-97-2); O21: Staurosporine, Streptomyces sp. (CAS 62996-74-1); P15: WHI-P180, Hydrochloride (CAS 211555-08-7); E13: Gö 6976 (CAS 136194-77-9); C09: Compound 56 (CAS 171745-13-4); A10: Alsterpaullone (CAS 237430-03-4); O03: AG 1478, Selective inhibitor of epidermal growth factor receptor (EGFR) protein (CAS 175178-82-2); N05: Reversine (CAS 656820-32-5). The chemical structure of these compounds is given in a Additional file 2.

Our methodology begins with the high-throughput screening of single drug and drug pair experiments. The 244 kinase inhibitors (KIs) of the EMD drug library were screened at 1000 nM individually and the treatment lasted for 72 hours. To quantify a selective response of a cancer cell line with respect to a control normal cell line, we define the selectivity *S* of a single drug or drug combination as

$$S=\frac{v_{N}}{v_{C}}$$

where *v*_*N*_ indicates the viability of normal cells (IMR90) after treatment, and *v*_*C*_ the viability of cancer cells (A549) after treatment. From the screening of the 244 KIs, the top hit was PDK1/Akt1/Flt3 Dual Pathway Inhibitor (CAS # 331253-86-2) as ranked by selectivity (Figure 1). For the secondary screen, we used the PDK1/Akt1/Flt3 Dual Pathway Inhibitor as the starting point and combined this compound with the other KIs as a drug pair combination. The dose of PDK1/Akt1/Flt3 Dual Pathway Inhibitor was studied to ensure proper dosing range and minimize toxicity. We used 125 nM, which maintains the normal cell line IMR-90’s viability >90% (Figure 2). For the other 243 KIs we used the standard dose of 1000 nM. Several pairs in the secondary screen showed very high selectivity. The top hit from the secondary screen of the library was Alsterpaullone 2-cyanoethyl (CAS # 852529-97-0) with a selectivity of S = 6.14 for the pair (Figure 3).

### Analysis of correlations

In our second step, we analyzed the Pearson’s correlation of the primary and secondary screening with a published dataset [4] containing target profiles for 140 kinase inhibitors. Therefore, even though we had a library of 244 KIs in the experimental screening, we were limited to utilizing 140 KIs for the analysis. For each inhibitor, the dataset provides the residual activity (0 ≤ *A* ≤ 1) of 291 kinases after drug treatment. This quantity is a measure of the strength of inhibition of a drug on each kinase.

For each kinase *k*, we calculate the Pearson’s correlation, *C*_*k*_, between the selectivity *S*_*i*_  and the activities *A*_*k*, *i*_, with *i* ∈ {1, …, *M*} indicating the single drug or drug pair in the set. For drug pairs, the activity is estimated as a product of the residual activities of the two drugs. The kinases are then ranked based on the *p-*value of their correlation with selectivity, and we calculate the False Discovery Rate (FDR) adjusted *p* value [12]. The list of kinases mostly correlated to the selectivity from the primary and secondary screen are listed in Table 1*.* We also did calculations of the correlation between the normal or cancer cell viability and the activities. The results for the top kinase-viability correlations for the primary and secondary screen are shown in the supplementary materials (Additional file 1: Table S1).

**Table 1:** Correlations between selectivity and kinase activity from primary and secondary screening

**Kinase**	**Selectivity corr**	**FDR**	**Kinase**	**Selectivity corr**	**FDR**
**Primary screening**			**Secondary screening**		
PRKCZ	0.451	2.28E-08	TGFBR2	-0.501	8.29E-08
DMPK	0.435	7.75E-08	CDK4	-0.412	6.40E-05
STK39	0.430	1.15E-07	CDC42BPB	-0.409	6.40E-05
EPHA8	0.420	2.33E-07	RIPK2	-0.399	7.73E-05
ADRBK2	0.399	1.01E-06	DSTYK	-0.369	0.000413
PRKACG	0.396	1.27E-06	ACVRL1	-0.368	0.000413
CAMK4	0.394	1.45E-06	PAK1	-0.367	0.000413
MAP2K2	0.393	1.53E-06	MAPKAPK2	-0.364	0.000413
ADRBK1	0.392	1.62E-06	PAK7	-0.359	0.000424
PNCK	0.382	3.29E-06	CDK1	-0.357	0.000429

A negative correlation indicates that inhibition of that particular kinases is associated to a higher selectivity. The top two hits with negative correlation, TGFBR2 and CDK4 are known to have an important role in cell proliferation, invasion and metastasis in lung adenocarcinoma [21,22].

### Elastic net regression

Next, we build a regression model that predicts the response of a cell line to a drug or drug combination *i.* The response we predict is the normal and cancer cell viability, from which the selectivity can be derived. For this purpose, we define a regression problem in which we use the residual activity of the kinase *k* under the effect of drug *i*, which we indicate as *A*_*k*, *i*_, as predictors of the viability. The response can be written as

(1) $$v_{i}=\beta_{0}+\beta_{1}A_{1,\;i}+\ldots +\beta_{p}A_{p,\;i}\;.$$

A fitting procedure based on a training set of measurements produces the coefficients (*β*_0_, *β*_1_, …, *β*_*p*_). Equation (1) can then be used to predict the viability of a new drug that has not been tested, but of which the profiling information is available. Note that we are integrating two different types of data: kinase profiling data is obtained through enzymatic assays that probe directly the interaction between drug and kinases, while the *in vitro* cell response data is the result of complex signaling that involves many pathways downstream of the affected kinases. The coefficients *β*_*k*_ can be seen as a measure of the sensitivity of a given cell line due to alterations in the activity of kinase *k*.

It is well known that the least square method does not perform well in the case of linear regression with many predictors. In our case, we would like to use a database of drugs that have been profiled on about 300 kinases. However, it would be desirable to select and keep in the final model a minimal set of the kinases that provide a simple model, useful to gain biological insight. The lasso technique [13] is a powerful method to reduce the number of predictors by imposing a penalty on the regression coefficients. However, in the presence of a group of kinase predictors with strong mutual correlation, the lasso could select only one kinase predictor from the group while missing the others. To prevent this problem, our method uses the elastic net approach. This method incorporates the lasso penalty as well as a ridge penalty to keep the regression coefficients small without completely removing them [6]. The weights of the ridge and lasso penalties in the least square procedure can be optimized for best performance of the method.

**Figure 4:**
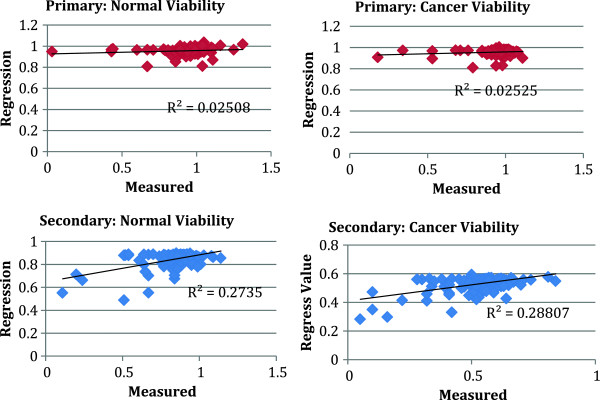
**Leave-one-out Cross Validation of the elastic net regression model based on the primary (top) and secondary (bottom) screens for normal and cancer cell lines.** Each of the 140 point in these figures corresponds to one of the 140 drug. “Regression” refers to the viability predicted by the regression model using all data from the other 139 drugs as training set, while “Measured” refers to the actual viability measured for the drug or drug combination. Note that only the secondary screen leads to predictive models with significant R^2^ for the two cancer cell types.

We show in Figure 4(a) and (b) the results of a leave one out cross validation (LOOCV) method for the primary (a) and secondary screen (b). For each of the 140 drugs, we apply the elastic net method using the remaining 139 drugs and then we compare the result to the measured value. This cross validation method is a particular case of the more general *k*-fold cross validation procedure in which *k* is equal to the size of the training set [14]. The cross LOOCV shows that the information contained in the primary screen is not sufficient to define a predictive model. The fact that some kinases in Table 1 show some significant correlation with the response when considered individually is in general not a sufficient condition for defining a predictive, multiple regression model. On the other hand, the secondary screen is able to reproduce the viability of many drugs, especially the ones with the stronger effect on both cell lines. Overall, the data from the secondary screen presents a much broader distribution with a tail representing a few drug combinations particularly effective. The regression works better in identifying these highly effective pairwise combinations and the relative ranking of their strengths. Data is not particularly informative for drugs and drug pair combinations that are not effective, which concentrate in the neighborhood of ~ 1.

Data transformations can represent a powerful strategy to improve regression. We applied a logarithmic transformation, which is consistent with the hypothesis of an independent action on the different kinases on the total viability. In this case we assume that the viability can be rewritten in the form

(2) $$v_{i}=e^{\beta_{0}}{\left(A_{1,\;i}\right)}^{\beta_{1}}\cdot {\left(A_{2,\;i}\right)}^{\beta_{2}}\cdot \ldots \cdot {\left(A_{p,\;i}\right)}^{\beta_{p}}\;.$$

**Figure 5:**
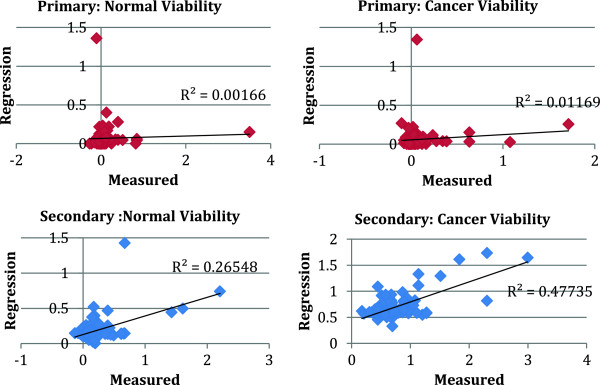
**Leave-one-out Cross Validation of the elastic net regression model based on the primary (top) and secondary (bottom) screens for normal and cancer cell lines after logarithmic transformation on the data.** Each of the 140 point in these figures corresponds to one of the 140 drugs. “Regression” refers to *–log* of the viability predicted by the regression model using all data from the other 139 drugs as training set, while “Measured” refers to *–log* of the actual viability measured for the drug or drug combination. Note that, as in Figure 4, only the secondary screen leads to predictive models with significant *R*^*2*^ for both cell types. The *R*^*2*^ for the Cancer cell lines is considerably better using the log transformation.

By applying a *log* transformation on both sides of Eq. (2) we reduce the problem to a linear regression, to which the elastic net strategy can be applied. We show in Figure 5 the results of the LOOCV for the primary and secondary screen using the logarithmic data transformation. As in the linear case, we find that the method fails the cross validation procedure if we use data from the primary screen, while the secondary screen with log transformed data gives better *R*^*2*^.

In addition to a regression model that can be used to predict the efficacy of drugs that have not been tested, the *β*_*i*_ coefficients can be used to rank kinases in terms of their relevance in the regression. Therefore, these coefficients identify the kinases whose inhibition is associated to a decrease in the cell viability. A ranking based on the differential $\beta_{i}^{C}-\beta_{i}^{N}$, where the index *N* and *C* identify the regression model of the cancer and normal cells, gives insight on specific pathways important for a selective response of cancer cells. Table 2 shows a list of kinases ranked in terms of $\left|\beta_{i}^{C}-\beta_{i}^{N}\right|$, where the coefficients have been obtained using the logarithmic data transformation on the secondary screen.

**Table 2:** Kinases with the highest difference in the regression coefficients for the log transformed data of the secondary screen

**Kinase**	**Cancer beta coefficient**	**Normal beta coefficient**	**Difference**
TGFBR2	0.061	0.000	0.061
EGFR	0.060	0.000	0.060
PHKG1	0.051	0.014	0.037
RIPK2	0.032	-0.002	0.034
PRKG2	0.012	0.045	0.033
CDK4	0.021	-0.008	0.029
MAP3K10	0.038	0.014	0.024
MARK4	0.000	0.022	0.022
PAK1	0.025	0.004	0.021
MAP4K5	0.021	0.000	0.021
MARK2	0.006	0.026	0.021
MARK3	0.000	0.020	0.020
TBK1	0.012	0.031	0.020
ERBB2	0.021	0.001	0.019
NUAK1	-0.029	-0.010	0.019
ULK2	0.018	0.000	0.018
MYLK2	-0.024	-0.006	0.018
MAP4K4	0.004	-0.014	0.018
CDK5	0.002	-0.016	0.018
GSK3B	0.021	0.004	0.017
PAK2	0.019	0.002	0.017
CDC42BPB	0.023	0.006	0.017
DSTYK	0.006	-0.010	0.016
RPS6KA2	0.000	-0.016	0.016
FGFR1	-0.004	0.012	0.016
PAK7	0.015	0.000	0.015
PIM1	-0.015	0.000	0.015
CDK3	0.015	0.000	0.015
IRAK1	-0.002	-0.017	0.015

A larger difference is associated with a selective response of A549 upon inhibition. Note that in addition to TGFB2R and CDK4, which were identified with the correlation approach of Table 1, additional kinases known to have an important role in lung cancer such as EGFR [24,25] and PHKG1 [26] are found using the elastic net approach.

In order to test whether selected pathways were significantly enriched for the identified kinase genes in Table 2, a pathway-based enrichment analysis was conducted using the results from the elastic net kinase analysis and Fisher exact tests. Ten pathways from Reactome were identified as significant (p < 0.05) using this kinase list, including axon guidance, activation of Rac, and semaphorin interactions as top hits (Table 3).

**Table 3:** Reactome pathways with significant representation of kinases from the regression analysis

**Path ID**	**Path name**	**N**_**S**_	**N**_**T**_	**p-val**
422475	Axon guidance	9	31	0.005
428540	Activation of Rac	3	5	0.008
373755	Semaphorin interactions	4	10	0.011
376176	Signaling by Robo receptor	3	7	0.024
1266738	Developmental Biology	8	39	0.026
445144	Signal transduction by L1	4	13	0.030
373760	L1CAM interactions	4	14	0.040
193639	p75NTR signals via NF-kB	2	4	0.051
209543	p75NTR recruits signaling complexes	2	4	0.051
389359	CD28 dependent Vav1 pathway	2	4	0.051

N_s_ indicates the number of kinases that are found significant in the regression analysis, while N_T_ is the total number of kinases in the pathway. The top ten pathways with Fisher exact test p < =0.051 are shown. These pathways are identified from 518. Reactome pathways containing at least one of the kinases identified in Table 2. The 9 kinases in the axon-guidance pathway are EGFR, PAK1, ERBB2, CDK5, GSK3B, PAK2, RPS6KA2, FGFR1 and PAK7.

## Discussion

Drug-kinase profiling represents a controller-target network [5] that when combined with *in vitro* testing, can be used in regression models to predict drug response and to identify pathways statistically associated to drug sensitivity. Network methods in biology are often based on the analysis of large datasets from high-throughput experiments. An example is given by gene regulatory networks, which presents many challenges either when restricted to a homogeneous set of data [15,16] or when it includes different classes of data [17-20]. In our KIEN method, information from the drug-target network and experimental query of the biological system are integrated. The goal is not a reconstruction of a regulatory network, but to identify a set of kinases linked to a therapeutic response in a given cell line. In order to establish associations, the system has to be perturbed by the use of kinase inhibitor drugs. The response to these single drugs or drug combinations becomes a training set that when combined with the kinase profiling, can lead to predictions.

The elastic net method is one of the most widely used regularization techniques. Regularization techniques are used in statistical and machine learning models to achieve an optimal tradeoff between accuracy and simplicity. Simplicity makes a model less prone to overfitting and more likely to generalize. In our analysis, we found that the elastic net regressions based on single drug responses were not successful, while drug pair data provided statistically significant predictions. A possible explanation for this finding is the following: single drugs might be less able to overcome the robustness of biological networks [5]. The phenotypic signal is therefore blunted and not easily measured. If a second drug is added, any compensatory capacity is already stretched and the effects from the inhibition of each kinase can be seen more clearly. Using data from drug pairs, we found that noise can be better filtered out and stronger statistical associations between kinases and therapeutic response are revealed. Clearly, if a different training set with higher variance in efficacy measures were used in the primary screen, it is likely that also single drug *in vitro* response would have given a significant predictive model.

We identified several kinases that are implicated in lung cancer that gives biological significance to our KIEN method. In particular, TGFBR2 appears as a top hit both in the correlation and in the elastic net methods. This finding is consistent with recent siRNA experiments on A549 cell lines [21], which demonstrated that silencing of this receptor reduces cell proliferation, invasion, and metastasis. The Cyclin-dependent kinase 4 (CDK4) appears as a second top target in the correlation analysis, and is also highly significant in the KIEN analysis. Experiments using lentiviral-mediated shRNA to inhibit CDK4 in A549 have shown inhibited cell cycle progression, suppressed cell proliferation, colony formation, and migration [22], and there is an ongoing clinical trial using a CDK4/6 inhibitor in lung cancer [23]. The KIEN analysis identified EGFR, which is known to be overexpressed in the majority of non-small cell lung cancers [24]. Furthermore, RNAi experiments targeting EGFR demonstrated cancer growth suppression in A549 xenograft in mice [25]. The third kinase in Table 2, PHKG1 has also been found to be upregulated in human tumor samples, including lung adenocarcinoma, and aberrations in its gene copy number is a feature of many human tumors [26].

The pathway-based enrichment provides a broader view on the role of the kinases identified by our method in Table 2. Among the top three pathways shown in Table 3 are activation of Rac and Semaphorin interactions. Rac proteins play a key role in cancer signaling and they belong to the RAS superfamily [27]. We also identified a set of semaphorins in our analysis that is represented in the top significantly enriched pathways. Semaphorins, previously known as collapsins, are a set of proteins containing a 500-amino acid sema domain among others (including PSI and immunoglobulin type domains), which can be transmembranous or secreted [28]. It is known that Sema3E cleavage promotes invasive growth and metastasis *in vivo*[28]. These genes also have selective targeting by Rac and Rho family members. This generates hypotheses of possible pathways that could be targeted therapeutically. However, these hypotheses need to be validated by further experiments with different inhibitors for the same targets or with alternative methods, e.g. using siRNA.

## Conclusions

We have introduced an integrated experimental and computational methodology that identifies the role of specific kinases in the drug response of a given cell line. The key element of our KIEN methodology is a multiple regression procedure that uses *in vitro* screen data as a training set. If a new library of kinase inhibitor compounds were to be synthetized and profiled, then our model would be able to immediately estimate the effect of these drugs on *in vitro* experiments on a given cell line. We have shown an application to a lung cancer cell line, but our method can be extended to different cell lines. The method will facilitate the design of new kinase inhibitors and the development of therapeutic interventions with combinations of many inhibitors [29]. The procedure could be extended to three drug combinations, if measurements for these larger combinations were available. Finally, the method could be extended to regression models that are specific of cancer cells with the same set of mutations, or it could be directly used with patient-derived primary cells to identify a personalized treatment.

## Materials and methods

### Materials

The primary screening of a kinase inhibitor (KI) library comprised of 244 KIs was purchased from EMD Chemicals, and diluted with DMSO to 2 mM concentrations for high-throughput screening purposes. The KI library was stored at -80°C. Additionally, PDK1/Akt1/Flt3 Dual Pathway Inhibitor (CAS # 331253-86-2) was ordered from EMD. Only 140 out of 244 were used in the drug-target network reconstruction because the drug profiling information was available only for these compounds. One kinase inhibitor known to affect the kinase targets indirectly was excluded. We provide in Additional file 2 the chemical structure of kinase inhibitors with highest selectivity in the primary and secondary screening.

### Cell culture

Cell lines IMR-90 (normal lung fibroblast) and A549 (lung adenocarcinoma) were cultured in RPMI 1640 (Hyclone) supplemented with 10% Canadian characterized fetal bovine serum (Hyclone), 1% 200 mM L-glutamine (Omega), and 1% penicillin/streptomycin (Omega). The media for the cells were renewed every 3 days and kept at 80-90% confluency. Cells were maintained in a humidified environment at 37°C and 5% CO_2_.

### Kinase inhibitor experiments

IMR-90 (1500 cells/well) and A549 (750 cells/well) were seeded on 384-well microplates (Grenier Bio-One) and incubated for 3 hours before the addition of kinase inhibitor(s). The reason that IMR-90 was seeded at double the cell density of A549 is due to the difference in cell division. IMR-90’s doubling time is 36–48 hours whereas A549’s is 22 hours. We wanted to make sure that the cells have divided at least once during the 72 hr drug treatment. Furthermore, both A549’s and IMR-90’s final confluency at 72 hrs is 90-95% and within the range of the ATPlite 1step assay. Additional file 1: Figures S1 and S2 show the growth curve for both cell lines. IMR-90 and A549 cell lines were tested on the same day with three replicates and the experiment was repeated three times with randomized well positions to reduce biases. ECHO 555 Liquid Handler (Labcyte) was used to dispense nanoliter volumes of each KI to 384-well plates with cells attached (wet dispense). The final volume in the plate is 40uL and cells were incubated for 72 hours with KI treatment.

### ATP measurements

ATPlite 1Step (Perkin Elmer) was used to evaluate the cell number and cytotoxicity. ATP measurements were done by dispensing 20 uL of the ATPlite 1Step solution to each well to a final volume of 60 uL. The plate was placed on a shaker at 1100 rpm and the luminescence activity was detected by Analyst GT Plate Reader. The percent (%) of control is the quantity of ATPlite 1step measurement of the treated versus the untreated wells of each individual cell type. The ATP standard was prepared with culture media to final volume of 40 uL, and 20 uL of ATPlite 1step reagent was added. Additional file 1: Figure S3 shows the ATP standard curve. The plate was read immediately.

### Computational methods

Correlations between selectivity/viability and kinase activity were calculated using the python *scipy linregress* function, which also provide *p-values.* Ranking the p-values and directly applying the Benjamini–Hochberg procedure gave us the FDR values. The elastic net regression was carried out using the Scikit-learn package [30] which finds the coefficients *β* that minimize the function

$$F=\frac{1}{2\;M}|\left|v-\mathrm{A\beta}\right||{}_{2}^{2}+\alpha \rho |\left|\beta \right||{}_{1}+\frac{1}{2}\alpha \left(1-\rho \right)||\beta ||{}_{2}^{2}$$

where *v* is the vector of the observed viabilities and *A* is the matrix containing the residual activity of the kinases from the profiling, and *M* is the total number of drugs or drug combinations used. The parameters *α* and *β* determine the relative weights of the lasso and ridge penalties quantified using *L*^1^ (|| |_1_ ) and *L*^2^ (|| ||_2_) norm, respectively. We used *α* = 0.15 and *ρ* = 0.01 in the results of Figures 4 and 5 and in Table 2. We also tried other values of these parameters, which did not give a significant difference in the results.

### Pathway-based enrichment

Reactome pathways were downloaded using a newer build of the 'biomaRt’ library (v2.12.0) in Bioconductor/R (v2.15.0). Gene symbols from the kinase list were converted to Entrez gene identifier numbers ('entrezgene’) and mapped against the gene ids in each Reactome pathway. For each pathway, the set of significant genes enriched within any given pathway was computed using a Fisher exact test. The procedure computes the significance (p-value) of observing significant kinases, as deemed significant by our method, within the selected pathway. These pathways are identified from 518 Reactome pathways. Given that our gene set consists entirely of kinases and would be generalized towards kinase-specific effects, the set of all kinases (~300) were selected for background adjustment and more sensitive enrichment of the pathways. This procedure was repeated for each pathway to generate p-values and pathway rankings. False discovery rate [FDR] values were later generated to further restrict significance.

## Competing interests

The authors declare that they have no competing interests.

## Authors’ contributions

GP and CP proposed the concept, EO and CP wrote the software and analysed the results, APH performed the Reactome analysis, TT performed all experiments, CP and GP. wrote the manuscript. All authors read and approved the final manuscript.

## Supplementary Material

Additional file 2 Chemical structure of drugs with the highest selectivity in the primary and secondary screen. Click here for file

Additional file 1 **Prediction of kinase inhibitor response using activity profiling, *in-vitro *** **screening, and elastic net regression.** Click here for file
