# Supplementary material for: Prediction of kinase inhibitor response using activity profiling, in vitro screening, and elastic net regression
Source: BMC Syst Biol. 2014 Jun 25;8:74. doi: 10.1186/1752-0509-8-74 (PMC4094402; doi:10.1186/1752-0509-8-74)
Supplement: Additional file 1 — Prediction of kinase inhibitor response using activity profiling, in-vitro screening, and elastic net regression. [file 1752-0509-8-74-S1.docx]

Supplementary Materials: Prediction of kinase inhibitor response using activity profiling, *in-vitro* screening, and elastic net regression

Supplementary Figure S1: Growth Curves of A549. Cells were seeded at three cell densities on 384-well microplates. The optimal density for A549 was 750 cells/well, as determined by achieving 90-95% confluency and being within the range of ATPlite 1step. The range of ATPlite 1step was defined using the ATP standard in the supplementary Figure 3.

Supplementary Figure S2: Growth Curves IMR-90. Cells were seeded at four cell densities. The optimal seeding density for IMR-90 was at 1500 cells/well, as determined by achieving 90-95% confluency and being within range of ATPlite 1step

Supplementary Figure S3: ATP Standard Curve. From the ATP standard solution provided in the kit, a 10-fold series dilution (1uM to 1pM) was performed in culture medium.

Supplementary Table S1: Correlation between viability of normal (top) and cancer (bottom) cell lines and kinase activity from primary and secondary screening.

| Kinase | Normal Viab Corr | FDR |  | Kinase | Normal Viab Corr | FDR |
| --- | --- | --- | --- | --- | --- | --- |
| Primary screening | | |  | Secondary Screening | | |
| ADRBK1 | 0.484 | 3.92E-07 |  | PAK1 | 0.653 | 6.23E-16 |
| DMPK | 0.481 | 3.92E-07 |  | PAK3 | 0.647 | 8.53E-16 |
| DDR2 | 0.459 | 1.15E-06 |  | PKN2 | 0.619 | 3.32E-14 |
| ZAP70 | 0.455 | 1.16E-06 |  | PDPK1 | 0.605 | 1.86E-13 |
| AKT2 | 0.453 | 1.16E-06 |  | SIK2 | 0.575 | 6.37E-12 |
| CAMK1G | 0.427 | 6.99E-06 |  | MAP3K10 | 0.568 | 1.22E-11 |
| TSSK2 | 0.428 | 6.99E-06 |  | FGFR2 | 0.562 | 1.98E-11 |
| MAPKAPK2 | 0.424 | 6.99E-06 |  | CAMK2G | 0.561 | 2.14E-11 |
| CAMK4 | 0.423 | 6.99E-06 |  | CDC42BPB | 0.559 | 2.20E-11 |
| PRKCZ | 0.419 | 7.54E-06 |  | NUAK2 | 0.545 | 9.97E-11 |

| Kinase | Cancer Viab Corr | FDR |  | Kinase | Cancer Viab Corr | FDR |
| --- | --- | --- | --- | --- | --- | --- |
| Primary screening | | |  | Secondary Screening | | |
| AKT2 | 0.549 | 6.01E-10 |  | PAK1 | 0.625 | 4.51E-14 |
| DMPK | 0.545 | 6.01E-10 |  | PKN2 | 0.567 | 4.33E-11 |
| CAMK1G | 0.528 | 1.87E-09 |  | PAK3 | 0.554 | 1.25E-10 |
| DDR2 | 0.506 | 1.35E-08 |  | MAP3K10 | 0.550 | 1.43E-10 |
| ZAP70 | 0.493 | 3.67E-08 |  | CDC42BPB | 0.539 | 3.81E-10 |
| CAMK4 | 0.488 | 4.50E-08 |  | MAP3K2 | 0.520 | 2.16E-09 |
| ADRBK1 | 0.477 | 1.07E-07 |  | PDPK1 | 0.519 | 2.16E-09 |
| SGK3 | 0.477 | 1.07E-07 |  | SIK2 | 0.519 | 2.16E-09 |
| GRK5 | 0.473 | 1.20E-07 |  | NUAK2 | 0.502 | 8.14E-09 |
| AKT1 | 0.454 | 5.09E-07 |  | FES | 0.502 | 8.14E-09 |
